# Commissioning of a mobile electron accelerator for intraoperative radiotherapy

**DOI:** 10.1120/jacmp.v2i3.2605

**Published:** 2001-09-01

**Authors:** Michael D. Mills, Liliosa C. Fajardo, David L. Wilson, Jodi L. Daves, William J. Spanos

**Affiliations:** ^1^ Department of Radiation Oncology University of Louisville 529 South Jackson Street Louisville Kentucky 40202

**Keywords:** intraoperative radiotherapy, machine commissioning, electrons, linear accelerator

## Abstract

Radiation performance characteristics of a dedicated intraoperative accelerator were determined to prepare the unit for clinical use. The linear accelerator uses standing wave *X*‐band technology (wavelength approximately 3 centimeters) in order to minimize the mass of the accelerator. The injector design, smaller accelerator components, and low electron beam currents minimize radiation leakage. The unit may be used in a standard operating room without additional shielding. The mass of the accelerator gantry is 1250 Kg (weight approximately 2750 lbs) and the unit is transportable between operating rooms. Nominal electron energies are 4, 6, 9, and 12 MeV, and operate at selectable dose rates of 2.5 or 10 Gray per minute. $D_{\max}$ depths in water for a 10 cm applicator are 0.7, 1.3, 1.7, and 2.0 for these energies, respectively. The depths of 80% dose are 1.2, 2.1, 3.1, and 3.9 cm, respectively. Absolute calibration using the American Association of Physicists in Medicine TG‐51 protocol was performed for all electron energies using the 10 cm applicator. Applicator sizes ranged from 3 to 10 cm diameter for flat applicators, and 3 to 6 cm diameter for 30° beveled applicators. Output factors were determined for all energies relative to the 10 cm flat applicator. Central axis depth dose profiles and isodose plots were determined for every applicator and energy combination. A quality assurance protocol, performed each day before patient treatment, was developed for output and energy constancy.

PACS number(s): 87.53.–j, 87.52.–g

## INTRODUCTION

The goal of intraoperative radiation therapy is to deliver a uniform single fraction dose of 10–25 Gray to a surgically exposed volume of tissue at risk (planning target volume), while the patient is managed under anesthesia. To treat the desired volume, it is necessary to assure a minimum surface dose, shield tissues not at risk, and vary the penetration of the radiation beam. For these reasons, megavoltage electrons are the modality of choice for treating patients with intraoperative radiation therapy. The shape and size of the radiation field may be controlled with an appropriate applicator selection and the use of lead shielding. Choosing an appropriate bolus thickness and selecting the electron energy, while allowing for the presence of the bolus, controls the entrance dose and beam penetration.

Until now, it has not been possible to perform intraoperative radiotherapy with electrons in a conventional operating room. Most often, the accelerators have been located within radiotherapy departments and patients have been transferred under anesthesia from the operating room to the accelerator room for treatment. In some cases, the entire surgical procedure was performed in a modified accelerator treatment room. In yet another approach, some institutions built an operating room specially shielded for a conventional electrons‐only linear accelerator.^1^ Each of these approaches have significant difficulties. The drawback of the first approach is the perceived increased risk of a serious accident or an infection while moving a patient over long distances and several floors with an open incision. The second option involves removing the patient and surgical team from the support of a dedicated operating room suite and absorbing the cost of retrofitting an accelerator vault with surgical lights, tables, gas lines, and other features necessary to perform surgery. In each of the previous cases, the accelerator vault must be scrubbed to receive a surgical patient and the unit must be closed to conventional radiotherapy for a number of hours. Unless the surgery is performed after normal clinic treatment hours, the scheduling of this activity can create significant problems for clinic operations. Intraoperative radiotherapy using conventional accelerators makes for inefficient use of expensive machines designed for high patient throughput. It is therefore inconvenient for both medical and technical staff, and cost ineffective respecting clinic operations. The final option involves the significant expense of adding sufficient shielding to the operating room to protect personnel from photon head leakage and in‐beam photon contamination. There is the additional loss of revenue during construction while the room is out of service. It is therefore desirable to develop new technology that allows a dedicated electron accelerator to be placed in a conventional operating room without the need for additional shielding.

## MATERIALS AND METHODS

IntraOp Medical, Inc. (Santa Clara, CA) has developed a mobile electron linear accelerator, the Mobetron® (Mobetron is a registered trademark of IntraOp Medical, Inc.), designed and configured for intraoperative radiotherapy.^2^ The Mobetron is a lightweight *X*‐band linear accelerator mounted on a *C*‐arm gantry. The gantry is attached to a stand that contains the accelerator cooling system and a transportation system. A mobile modulator rack, a lightweight operator control console, and connecting cables complete the Mobetron system. The Mobetron may be adjusted for two configurations: accelerator horizontal with a low center of gravity for transportation and storage; and accelerator vertical for treatment. In transport configuration, the Mobetron is compact; the dimensions are such that it may fit on many elevators. The unit can be removed from the operating room for maintenance and annual calibrations. The control system contains the dosimetry readout parameters, accelerator controls, machine interlock status, and a color video output of the treatment viewing system.

The Mobetron produces electron beams of nominal energies 4, 6, 9, and 12 MeV. The gantry is in the configuration of a *C*‐arm, but with some additional flexibility of movement. The gantry may be rotated ±45° downward in the transverse plane. In addition, the gantry may be tilted ±30° in the radial plane. Also, the gantry may be moved in and out, and from side to side in the horizontal plane, ±5 cm. The gantry tilt and horizontal movements are unique features not found in conventional accelerators used for intraoperative radiotherapy. The axis of rotation is 99 cm (39 inches) above the floor and the nominal electron source to treatment surface distance (SSD) is 50 cm (i.e., 50 cm to the end of the treatment applicators). Gantry rotation and tilt movements are controlled from the hand held pendant and are variable from 0 to 1° per second. Horizontal movements are controlled in a similar manner and vary from 0 to 2 mm per second. The gantry design includes an integral beamstop to intercept photon contamination generated in the accelerator, collimation system, and the patient. The gantry with accelerator, cooling system, beamstop, and transportation system has a mass of 1250 Kg (weight approximately 2750 lbs). Mobetron transportation is accomplished by using a modified pallet jack, located at the rear of the gantry stand. Wheels attached to the front of the gantry support legs and wheels integral to the pallet jack provide a stable support for transportation.

The transportability and positioning flexibility of the Mobetron is made possible by the *X*‐band accelerator design. While conventional medical linear accelerators operate in the *S* band (10 cm wavelength, 3 GHz frequency), the Mobetron operates in the *X* band (3 cm wavelength, 10 GHz frequency). The diameter of the accelerator structures is therefore reduced by a factor of three.

Overall, the smaller accelerator structure and associated electronics leads to a considerable reduction in weight, compared to conventional accelerator technology. This weight reduction is required if the accelerator is to be mobile.

The Mobetron uses two *X*‐band linear accelerators in tandem. One‐third of the radio frequency power is injected into the first accelerator, producing electron energy of 4 MeV. The remaining two‐thirds of the power may be absorbed in a water load and/or injected into the second accelerator guide. Adjusting the phase to change the amount of power that enters the second guide, as opposed to the water load, varies the energy. As the power in the second guide is changed, the phase of the microwaves in the second guide is simultaneously adjusted to maintain optimal resonance in the accelerator structure. This allows energy control between 4 and 12 MeV without using a bending magnet. The injector system, together with a prebuncher and beam alignment system, control the electrons to occupy a very narrow energy spectrum, reducing radiation leakage. Since the bending magnet is a major source of leakage radiation in conventional accelerator designs, this design feature also contributes to a significant reduction of photon leakage. As the unit is designed to operate only in the electron mode, beam currents are low, producing less inherent radiation leakage. Together with the compact beamstop opposite the electron beam, the overall design allows the system to be used in rooms with no additional shielding.^3^

The Wellhöffer RFA‐300 Beam Scanning System and WP‐700 software (Wellhöffer North America, Bartlett, TN) was used for radiometric measurements and film analysis. Film dosimetry was accomplished using Kodak XV‐2 film (Eastman Kodak Co, Rochester, NY). Films were scanned using a Vidar VXR‐12 film scanner (Vidar Corporation, Herndon, VA). Central axis scans were performed with the PTW T23343 Markus chamber, while absolute dosimetry was accomplished using the waterproof PTW T30006 Farmer chamber and the PTW T34001 Roos Chamber (PTW New York Corporation, Hicksville, NY). The electrometer used for absolute calibration was the CNMC Model 206 (CNMC Company, Inc., Nashville, TN). One‐dimensional beam positioning, for calibration and output factor determination, was accomplished using the Accucal 1D positioning system (Gammex RMI, Middleton, WI).

Mobetron measurements were performed in a patient changing area, temporarily dedicated for this purpose. The corridors and adjacent rooms were cordoned off to define a controlled area for radiation safety purposes. Beam measurements were performed after hours to assure only occupationally exposed personnel would accrue any exposure associated with the commissioning process. Radiation safety considerations for personnel limited the machine‐on time to approximately 30 minutes (30,000 monitor units) per week. The proper position of each applicator was assured in the commissioning process by using a specially designed applicator support that attached directly to the accelerator head. This applicator support cannot be used during patient treatment, otherwise the sterile surgical field would be compromised.

The commissioning process resulted in a daily program for quality assurance (QA). The Mobetron is warmed up each morning before surgery and dosimetric checks are performed, assuring consistency of output and energy. The results of the QA check are recorded in the machine logbook, which is analyzed periodically to determine any potential trend for change in output or energy.

Using the Mobetron for patient treatment requires a sterile treatment applicator to be placed directly in contact with the volume to be treated, as determined by the radiation oncologist. The applicator is held in place using a sterile clamp that is attached to the surgical table. The patient, with the applicator clamped in place, is moved beneath the Mobetron gantry. Docking is then completed without the applicator contacting the Mobetron, so preserving the sterile field. The accelerator position is adjusted until a laser controlled interlock system indicates the electron beam is accurately aligned along the axis of the treatment applicator. A television monitor allows the radiation oncologist and surgeon to view the patient treatment surface area from the control unit.

The control unit for the Mobetron is located in a controlled‐access hallway adjacent to the operating room. Once the Mobetron is aligned, all personnel leave the operating room during patient treatment. Portable video display monitors allow the anesthesiologist to monitor the patient parameters from the hallway during treatment. Treatments of 12 to 20 Gray typically take 2 minutes to complete.

## RESULTS

Central axis scans were performed for each energy and applicator combination using the Wellhöffer system. The Markus chamber was chosen for central axis scans; the advantages of the Markus chamber include a small collection volume and knowing with precision the location of the proximal electrode, and thus, the point of measurement. The chamber was positioned along the central axis for all depth scans. Depth‐ionization measurements were determined with the axis of the applicator perpendicular to the water surface for all applicators. Flat applicators were positioned such that the applicator end just touched the water. A scan was first performed on the flat applicator; then the beveled applicator of the same size was scanned. Beveled applicators were covered with a clear thin plastic bag held in place with a rubber band. As with the flat applicators, the axis of the beveled applicators was perpendicular to the water surface. The water level was raised to top of the bevel; depth was assigned by matching the percentage depth dose of the beveled applicator to the depth dose profile of the corresponding flat applicator. The water was then lowered to the initial level, and scans for the 10 cm applicator were repeated to determine the degree of uncertainty in our measurements. This procedure indicated a precision of approximately ±0.5 mm to reproduce the measurement depth with the Markus chamber. Ionization was converted into dose using the Wellhöffer WP‐700 software that is based on the AAPM TG‐25 Report methodology.^4^ Results from the dose conversion were then used to determine the required information for the absolute dose calibration of the 10 cm flat applicator using the AAPM TG‐51 protocol.^5^ Output factors were then determined for each individual applicator and energy using the $D_{\max}$ values reported by the WP‐700 software for each scan.

Electron penetration characteristics are shown in Table I for the 10 cm flat applicator. The output factor is defined as the ratio of the dose measured at $D_{\max}$ for a given applicator and energy, relative to the dose at $D_{\max}$ for the 10 cm applicator and the same energy. A complete list of output factor values for the standard applicator set is given in Table II. We note a discontinuity of output factor values; applicators from 7 to 10 cm have a slightly different design than applicators 3 to 6 cm. Reproducibility of output factor values was determined to be ±0.5%. Information from central axis scans for the 10 cm flat applicators is used as input into the TG‐51 calibration methodology. Equation (6) from AAPM TG‐51 report calculates the dose from an electron beam,

(1) $$D_{W}^{Q}=MP_{gr}^{Q}k_{R50}^{\prime }k_{\text{ecal}}N_{D,w}^{60\;\text{Co}}(Gy).$$

$D_{W}^{Q}$ is the dose to water for beam quality *Q*; *M* is the fully corrected ion chamber reading; $P_{\text{gr}}^{Q}$ is the gradient correction factor; $k_{R50}^{\prime }$ is the electron quality conversion factor determined by $R_{50};k_{\text{ecal}}$ is a chamber‐dependent factor required to convert the chamber calibration factor $N_{D,w}^{60\;\text{Co}}$ into an electron beam absorbed‐dose calibration factor for a selected beam quality. The chamber placement reference depth for electron beam dosimetry is at $d_{\text{ref}}=0.6\;R_{50}-0.1\;\text{cm}$. Input and calculated values are given below in Table III for the absolute calibration of the four electron energies using the PTW 0.6 cc waterproof Farmer replacement chamber. This chamber was used to calibrate all electron energies of the Mobetron for clinical service. However, the TG‐51 protocol recommends the use of a parallel plate chamber for electron beams with $R_{50}$ less than or equal to 4.3 cm. Consequently, the Roos parallel plate chamber was also used to calibrate each of the electron energies, and the results of these calibrations were compared. Calibrations using each of these chambers agreed within 0.5% for the 9 and 12 MeV energies, and within 1% for the 4 and 6 MeV energies.

**Table I:** Mobetron depth for a given percentage of $D_{\max}$ dose values for a 10 cm applicator.

Depth (cm) to	Energy			
	4 MeV	6 MeV	9 MeV	12 MeV
$D_{\max}$	0.76	1.39	1.98	2.06
90%	1.08	1.94	2.92	3.50
80%	1.24	2.15	3.24	3.93
50%	1.54	2.63	3.89	4.77
30%	1.75	2.92	4.27	5.28

**Table II:** Mobetron output factor values for flat and beveled applicators.

Applicator (cm)	Energy			
	4	6	9	12
10	1.00	1.00	1.00	1.00
9	1.016	1.017	1.023	1.046
8	0.963	1.043	1.050	1.070
7	0.965	1.005	1.050	1.096
6	1.044	1.085	1.159	1.172
5	0.954	1.064	1.162	1.195
4	0.845	1.001	1.123	1.202
3	0.661	0.861	1.098	1.212
6/30° bevel	0.958	1.027	1.105	1.113
5/30° bevel	0.853	0.961	1.063	1.150
4/30° bevel	0.767	0.889	1.009	1.110
3/30° bevel	0.588	0.770	0.996	1.150

**Table III:** Selected PTW‐0.6 cc chamber correction factors for the calibration of electron dose using the TG‐51 protocol.

Factor	Energy			
	4 MeV	6 MeV	9 MeV	12 MeV
$P_{\text{gr}}^{Q}$	0.992	0.992	0.997	0.995
$k_{R50}^{\prime }$	1.037	1.025	1.016	1.009
$k_{\text{ecal}}$	0.897	0.897	0.897	0.897
$d_{\text{ref}}(\text{cm})$	0.82	1.48	2.23	2.76

The venue for commissioning imposed some limitations on how much beam time would be allowed during the commissioning process. Machine‐on time was limited to approximately 30 minutes per week. With this limitation, we could not obtain isodose curve data using the conventional methodologies of the Wellhöffer beam scanning system. We decided instead to obtain this data using a film scanning methodology. Isodose curves were generated directly from film using the following data conversion methodology: the electron films were scanned using the Vidar scanner with the Wellhöffer data acquisition software. The resolution of the scanner is twelve bits, and the density values are stored as sixteen bit integers in a raw data file. Normally, this file is exported directly to the Wellhöffer software for data analysis, but in this study we included an extra step to correct for film nonlinearity with dose.

**Figure 1:**
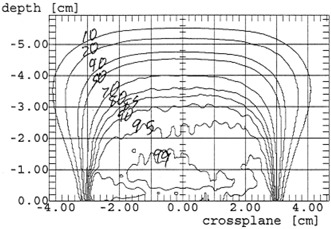
12 MeV isodose plot for a 6 cm flat applicator using film.

**Figure 2:**
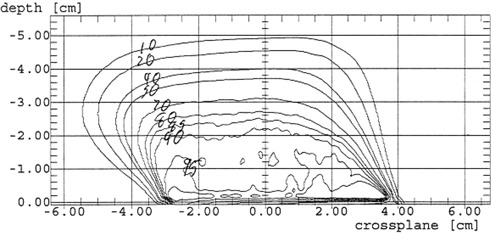
12 MeV isodose plot for a 6 cm, 30° beveled applicator using film.

To generate a film response curve, Kodak XV2 films were exposed to electron beam doses of 1, 5, 40, 60, 80, and 100 cGy. Those films were scanned, and a sample of density readings made near the center of those films was averaged for each dose range. A plot of dose versus density was fit to a quadratic formula and the coefficients of this fit were renormalized so that the first order term is unity. The films used for electron isodose determination were from the same box as the calibration films and were developed and scanned during the same session. A small program was written to process the raw density files. Those files contained 2638 bytes of header data, which was read and then written to an output file. The remaining data values were then read one at a time and the background density value was subtracted. Those numbers were put into the renormalized quadratic equation to generate new values that reflect actual dose response when compared one to another. Since the new numbers will be larger than the original values, the new integers were halved to ensure they would not exceed 4095. As expected, comparing the input values and the converted values demonstrated the same relative response as the dose‐density curve, indicating the conversion to dose was being performed properly. The sixteen bit integers were written one at a time to the new output file. This output file was then exported to the Wellhöffer WP‐700 software for isodose curve plotting. Central axis depth dose information obtained from the film was compared to that obtained from the ionization chamber using the Wellhöffer beam scanning system. Central axis depth dose values obtained using the Markus chamber were found to agree within ±1 mm for depths between the surface and $D_{50}$. Examples of plots for flat and beveled applicators are given in Figs. 1 and 2. Although the isodose curve data is not as smooth as would be obtained using conventional methodologies, the plots are considered sufficiently accurate and precise for clinical use.

Our experience with conventional intraoperative therapy and subsequently with the Mobetron system leads us to conclude that for most every patient presentation, it is possible to position the patient in direct contact with the electron applicator without a significant air gap. Air gap corrections are therefore a rare clinical requirement and to this date have not been utilized in our clinic. Nevertheless, we investigated the change in output with air gap, for gaps up to 1 cm. A linear equation to modify the output factor is given by,

(2) $$OF_{\text{gap}}=OF_{50}{\left[50/(50+\text{gap})\right]}^{2},$$

where the gap may be any value between 0 and 1 cm. We found this equation to result in a correct output factor within ±3% for each applicator/energy combination.

**Figure 3:**
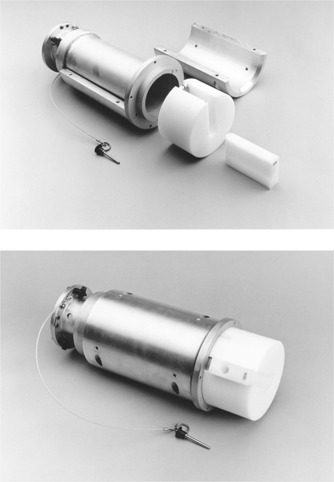
Polyethylene cylindrical phantom attached to a 10 cm applicator.

Before the Mobetron may be used clinically, it must undergo a warm‐up and quality assurance procedure. This is normally performed in the early morning before preparation of the operating room for surgery. A polyethylene cylindrical phantom attached to a 10 cm applicator and used for output and energy checks is pictured in Fig. 3. A rectangular slot cut into the cylinder accepts an insert drilled for a Farmer‐type chamber and buildup cap. The Cobalt‐60 energy buildup cap for the PTW‐Farmer chamber was machined to fit into the holes drilled in the inserts. This chamber was dedicated for use only in the Mobetron. Four inserts were drilled to accept a chamber at the $D_{\max}$ depth, corresponding to each of the four electron energies. An additional four inserts were drilled at a depth beyond $D_{80}$ for each electron energy and corresponding to a point on the steeply sloping part of the depth‐dose curve. The ratio of the ionization, measured in the latter insert to that of the $D_{\max}$ insert, is a sensitive measure of energy and reveals any shift in beam penetration that would be clinically significant. Phantom factors for the $D_{\max}$ inserts were determined at the time of initial electron output calibration. Energy check ratio standards were determined along with corresponding checks of $D_{80}$ and $D_{50}$ in water, to assure these standards correspond to commissioning data. After over 20 warm‐up procedures, output was found to be within 2% for all energies on almost every occasion. Twice, for two different energies, the dose output needed adjustment to fall within 2%. Energy ratios were found to fall within 5% on every occasion. This corresponds to an isodose shift of 0.5 mm or less. The warm‐up worksheet we use is presented in the Appendix.

A radiation safety survey was performed after hours for the two operating rooms that are used with the Mobetron. The maximum radiation exposure allowed for a specified energy for one week was determined for each of the four energies. From this exposure, we determined the maximum dose at $D_{\max}$ and the maximum number of monitor units that could be generated in a week for each energy, if only that energy were activated. Regulation limits are 0.02 mSv in any hour assuming full occupancy, and 0.02 mSv per week, assuming appropriate occupancy factors.^6^ The maximum monitor units are presented in Table IV.

**Table IV:** Workload limits including warm‐up per week, for two operating rooms by energy.

		Operating room 5			Operating room 8	
Energy	Warm‐up monitor units	Treatment monitor units	Total MU inc. warm‐up	Warm‐up monitor units	Treatment monitor units	Total MU inc. warm‐up
4	1200	4300 or	5500 or	1200	4200 or	5400 or
6	1200	6800 or	8000 or	1200	5300 or	6500 or
9	1200	4800 or	6000 or	1200	4300 or	5500 or
12	1200	4300	5500	1200	4300	5500

These energy workloads may be combined by percentage, assuming the total does not exceed 100%. For example, 25% of the workload of 6 MeV + 50% of the workload of 9 MeV + 25% of the workload of 12 MeV is allowed under current radiation regulations. All warm‐up, patient treatment, and maintenance monitor units are recorded in a logbook to assure these limits are not exceeded. A typical warm‐up uses 400 monitor units per energy, and a patient usually has one or two sites treated. Typically it is possible to treat two patients per week in each operating room before exceeding regulatory limits. The total exposure per week at the console is less than 2 mrem.

## CONCLUSION

Using the Mobetron intraoperative radiotherapy system, it is possible to treat up to four patients per week in our operating room suite with megavoltage electrons. This may be accomplished using standard physics and dosimetric techniques, while maintaining the highest standards for patient and personnel safety. Regulatory limitations of 2 mrem per week for personnel are met, without the requirement of additional construction and shielding in the operating rooms.

## ACKNOWLEDGMENTS

The authors appreciate the kind assistance of Michael Kelly, MS, Radiation Safety Officer of the University of Louisville. In addition, the authors wish to recognize the kind assistance and support of Tom Cook, Manager of Service Operations for IntraOp, Inc.

## Appendix

**MOBETRON IORT Output and Energy Check Date:**_____ **OR#**_____

**I Set up:**

SF6 pressure before _____ and after _____ recharging.

Turn circuit breakers on and press reset. Turn power key on, press clear to clear screen, press reset+beam interrupt for system reset. Wait 10 minutes, count down to complete

Mag Htr_____ Gun Fil_____ Vacuum _____

Set gantry and tilt t vertical, attach QA phantom to collimator head, attach lead shield to QA phantom, insert the QA key into it's slot, insert $d_{\max}$ block for 4 MeV into QA phantom.

**Table:**

**II**	**Output Check**	PTW N23333 Acrylic Chamber
		CNMC 206 Electrometer
		IntraOp Polyethylene QA phantom

Readings for 200 MU, 1000 MU/min, insert ion chamber into QA block, take 1–2 readings, Replace dmax block with d50 block for energy E, insert chamber into block, and take 1 reading. Acceptable values are ±3% for dose and ±5% for energy checks.
